# New Sources of Income for the Hospitals

**Published:** 1887-12-17

**Authors:** 


### New Sources of Income for the Hospitals.

By the Duke of Argyll.

It was determined by those who are connected with the management of the London hospitals that there should be a special effort to increase the income of all these institutions from voluntary sources during this year of Jubilee. In these circumstances I very readily assented to the request that I should put before you the main facts. I think the first thing we have to consider is the magnitude of the work we have to do, arising from the enormous population of the city in which we live. The population of London, as I dare say many of you know, is estimated at this moment to be somewhere about four million people, and before the next census is taken it is almost certain that it will considerably exceed that enormous number. Now, there is no reason to believe that, in any other country or age in the world, there has ever existed such a vast aggregate of human population in any one city. We hear in ancient history of the great cities of Nineveh and Babylon, but the accounts of those great cities have come to us through an exaggerating atmosphere of tradition. I think we may be perfectly sure, from the condition of society in the ancient world as compared with that which exists at the present day, that those vast cities—the greatest of the ancient world—did not approach the population of the city in which we live. Whatever may have been the case with the ancient world, there is now no other city of such magnitude. The largest city next to London is Paris, and the population of that city is a great deal short of that of London. The city of New York is next in population, and it is little more than a third of London. I never drive through the streets of London without feeling astonished at its vastness, and I ask myself how it is possible that this great mass of population can be provided with the necessaries and the means and the luxuries of life. There is no organization in London. The fact is that every man pursues his own interests, and when every man pursues his own interests we find he is pursuing the interests of the whole community. There is an old proverb that Rome was not built in a day. London was not built in a day. The whole secret of the accommodation of the population of London is that it is a gradual growth. No organization is necessary to feed London nor to supply the other necessities of civilized life. But we cannot say the same with regard to the provision for sickness and disease. We are here confronted with one of the most difficult questions of modern philosophy and politics. What are the things that can be left to individual interest and are thereby amply provided for, and what are those other things which absolutely require the intervention of organisation, either voluntary, or municipal, or State organisation? Well, we may answer without doubt that the provision for the cure of sickness and disease cannot be left to the individual interest of individual men. Death comes to all of us; it is not equally certain that disease will come to all of us. Death may come without disease, or without long and lingering and painful disease, and most men hope that death will come under these circumstances. I remember reading a book written by a most eminent philosopher, who said that if it were not for some habits of life which violate every rule of sanity and virtue, death would come to all men as it comes to many of the lower animals, as a leaf or ripe fruit falls to the ground. Probably some of us have known individual cases in which this has been the actual mode in which men have gone out of the world. I remember a very remarkable case of a friend of mine, who lived to be ninety-five, and who expired from mere *lapsus*. But, alas! this is not the fate of most men; it is the happy lot of a very, very small number, and therefore we must provide for sickness—long and painful sickness—afflicting a great part of the whole race. Now, what is the organisation with which we must meet this great necessity? Hitherto it has been entirely voluntary organisation, with the exception of the infirmaries, which are included under the Poor Law. There are no hospitals which are supported by municipalities or by the State. They all depend upon voluntary contributions; some, to a limited extent, upon property; but that property has been derived from donations and subscriptions of deceased benefactors. Practically the whole hospital accommodation depends entirely upon voluntary subscriptions. Now, how has that duty been performed? I am sorry to say, from the figures before me, that it has been very imperfectly performed, not only in London, but all over the kingdom. The hospitals of London are living from hand to mouth; they are obliged to draw upon their property in some cases, if not to close their wards That is the evil before us. Pray let us remember that although we are accustomed to consider the hospitals as for the relief of persons comparatively poor—most members of society being treated at home in their own airy houses—we are apt, I say, to consider that the hospitals are not for those who are in ample circumstances; but this is by no means true. We forget that the skill and knowledge applied in our case is the experience and the knowledge which have been acquired in the wards of hospitals. There is not a single medical man who would tell you it would be possible to rear a medical profession with knowledge derived from books alone; clinical teaching, that is, teaching by the bedside, is absolutely necessary. It is, therefore, an organization which should embrace all classes. Now, to return to London. London is by no means an unhealthy town. It stands very high in the death-rate statistics of the country. Its average annual death-rate per 1,000 living persons is 19; Manchester is 26—very much less healthy; Dublin is 28; Glasgow is 25·8; and Brighton alone of our large towns is lower than London, being 17 per 1,000. But, nevertheless, although London is a comparatively healthy town, we have only to consider what that death-rate means in a year; it is a striking, an awful, a solemn thought, that that death-rate produces the great army of 80,000 men, women, and children who are every year passing silently through the gates of death in this city. Yes, 80,000 men, women and children are annually dying, producing a total death-rate of no less than 219 persons a day. Now, to that death rate what is the proportion of serious diseases? I do not know whether anyone can inform me what is the proportion of persons who are under serious affliction from disease in proportion to those 80,000 deaths, but I apprehend if we multiply by five or six, we shall be below the figure. No less than one million cases were dealt with in the hospitals of London last year, representing a quarter of the total population of the city. You see therefore, what an enormous mass of suffering and disease you have to deal with and for which it is your duty to provide. I am sorry to say that in London the provision is insufficient. The total expenditure of the hospitals is £400,000 a-year, the total income, including that derived from property, is a little more than £300,000, leaving a deficit of £100,000. In this particular part of the city with which we are connected—which by-the-by is one of the largest, most populous and most wealthy in London—I am happy to say we stand very high in the contributions which are made towards the hospitals; and we ought to do so, because it is perhaps the richest community on the face of the globe. But still they fall short of the necessities of the case by the very considerable sum of no less than £18,000 in the year, and that counting all the proceeds derived from property. If we were to take alone the contributions from charity, the deficit would be very much larger—something like £40,000 a-year; but including property, dividends which are received from property, and the fees received from patients, the deficit is reduced to £18,000 The question is, How is this deficit to be made good, and how are we to succeed in establishing a greater and better organised effort for the support of these invaluable institutions? Perhaps you will allow me to refer to some information from my own country, the west of Scotland. Glasgow has an enormous population—upwards of half-a-million. It is not a very healthy city, with a very wet climate and occupations not of the healthiest kinds. It is pretty well provided with hospitals, but, just like the hospitals here, they have an annual deficit, and they have been obliged to consider with care how to remedy the evil. They have adopted a plan which promises very great results. They have determined, besides appealing to the Christian churches, to make a purely secular appeal to the industrial and working classes, because these to a very large extent benefit by the help of these hospitals. I should like to give you a few figures, for they are extremely interesting, but before giving these figures I ought to refer to the question of the hospitals being supported by the rates. A gentleman whom I know, from Glasgow, has written a very able paper in a journal devoted to the consideration of sanitary matters, the *Sanitary Journal,* in the argument of which I perfectly agree. He says that to provide for the deficit in the city of Glasgow you require an additional rate of threepence in the pound, and he points out that rates of this kind cannot be paid by the whole of the community. Ratepayers to a very large extent are poor men, and there are a very large number of ratepayers in this town, and in all towns, such as small shopkeepers, to whom an addition of threepence in the pound to their rates would be a most serious burden. Then we ought to remember that the moment we appeal to the rates, we put an end to voluntary subscriptions and donations. Therefore, although the threepence is the calculation of my friend in Scotland, as being enough to supply the deficit, the deficit would be multiplied in the very operation, and the whole expenses—not the deficit of the hospitals—would be thrown upon the rates. That would be a fatal result—I think a most unjust one. It is not only the owners of real property upon whom rates invariably fall. It is upon all classes of the community, and if you put a stop to voluntary contributions you put the rates upon a small class of the community alone. It would also be a great blow to the Christian character of the community if the hospitals were not to be continued by voluntary contributions. Glasgow is a very large manufacturing city in the centre of a great industrial district, full of collieries, of great shipbuilding yards, and other public works. Well, it occurred to the friends of hospitals to approach the working classes through their employers. They suggested to the employers that they should call their men together, and ask them to lay by a small percentage of their wages, to be fortnightly deducted for the purposes of the hospitals, and in return for that sacrifice on their part it was resolved to consult their feelings and opinions, and to allow them a voice in the dispensing of the fund thus accumulated. The result has been this, I am sorry to say it is not very creditable to the Christian churches that this secular appeal to the industrial classes has been immensely more successful than the appeal to the churches, producing a far larger sum; and the movement is yet in its infancy. The Glasgow Infirmary received last year from the collections in the churches £857; but from the industrial classes through this movement that infirmary received £4,868. The Western Infirmary—the second largest—received from the churches last year £1,105, and from the industrial classes £2,985. I observe that it is said that the industrial classes in London hardly contribute anything. But that is because they have not been appealed to in a systematic manner. There is a want of organization, a want of knowledge how to do it. Education is growing in this country, and if a general appeal were made, you would, I believe, have a much larger collection from the industrial classes than you would on Hospital Sunday. It may, perhaps, be said that there are no workshops in London exactly comparable to those in Glasgow. London is an aggregate of the agencies of distribution. Everything comes to London to be redistributed; but if you look into the list of subscribers in Glasgow, you will find it is not merely the great shipbuilding yards, it is not merely the collieries, but the large shopkeeping establishments in the city of Glasgow which contribute to a very large extent. Now, for every large shop in Glasgow there must be at least a hundred large shops in London, containing a very large number of employés. Individual shops in Glasgow contribute £12, £15, £20, and £40 a-year. There is another source of supply from the industrial classes, and that is from the great railway stations. In Glasgow there are practically only two great railway companies with very important stations—the Caledonian Railway Company and the North British Railway Company, and one or two minor companies. Now, these are not comparable with the great London companies. But I find that the workmen—I do not mean the officers of the companies—employed in the different departments gave last year, one company £113, and the other £120. This was, I think, a most satisfactory result. I hope very earnestly that those who are connected with this movement in London will copy the example set in Glasgow, and set on foot an organisation among the employers of all classes of labour, and that they will suggest to their workmen to agree to contribute a small sum fortnightly, and if this is done you will add a very large sum to our Hospital Sunday Fund. I feel sure that if this plan were adopted in London there would soon cease to be any deficit whatever. There are some curious facts in reference to the selection of particular hospitals which the working men support; but if you ask men to subscribe, it is but fair that they should have a voice in the disposal of their money. The disposal of the funds collected from the working classes ought to be regulated as far as possible by their wishes and desires, though they may sometimes make curious selections. In Glasgow they are particularly fond of subscribing to the Ophthalmic Hospital, although I do not know that ophthalmia is a very prevalent complaint in Glasgow; and as regards the Ophthalmic Hospital, the subscriptions are largely in excess of all other institutions put together. Those who subscribe are allowed a certain number of tickets for patients, and that gives them a personal interest in the results of their own subscriptions. I have now laid before the public the main facts of the case, and I leave it to them to take such steps as may be necessary to organize their offerings to the hospitals of London on the plan which has proved so successful in Glasgow.

